# Genomic Determinants of Pathogenicity and Antimicrobial Resistance for 60 Global *Listeria monocytogenes* Isolates Responsible for Invasive Infections

**DOI:** 10.3389/fcimb.2021.718840

**Published:** 2021-10-27

**Authors:** Dawei Shi, Tanveer Muhammad Anwar, Hang Pan, Wenqin Chai, Sihong Xu, Min Yue

**Affiliations:** ^1^ Division II of In Vitro Diagnostics for Infectious Diseases, Institute for In Vitro Diagnostics Control, National Institutes for Food and Drug Control, Beijing, China; ^2^ Institute of Preventive Veterinary Sciences & Department of Veterinary Medicine, Zhejiang University College of Animal Sciences, Hangzhou, China; ^3^ Zhejiang Provincial Key Laboratory of Preventive Veterinary Medicine, Hangzhou, China; ^4^ State Key Laboratory for Diagnosis and Treatment of Infectious Diseases, National Clinical Research Center for Infectious Diseases, National Medical Center for Infectious Diseases, The First Affiliated Hospital, College of Medicine, Zhejiang University, Hangzhou, China; ^5^ Hainan Institute of Zhejiang University, Sanya, China

**Keywords:** *Listeria monocytogenes*, listeriosis, genetic diversity, virulence factors, antimicrobial resistance

## Abstract

*Listeria monocytogenes* remains a significant public health threat, causing invasive listeriosis manifested as septicemia, meningitis, and abortion, with up to 30% of cases having a fatal outcome. Tracking the spread of invasive listeriosis requires an updated knowledge for virulence factors (VFs) and antimicrobial resistance features, which is an essential step toward its clinical diagnosis and treatment. Taking advantage of high-throughput genomic sequencing, we proposed that the differential genes based on the pathogenomic composition could be used to evaluate clinical observations and therapeutic options for listeriosis. Here, we performed the comparative genomic analysis of 60 strains from five continents with a diverse range of sources, representing serotypes 1/2a, 1/2b, 1/2c, and 4b, comprising lineage I and lineage II and including 13 newly contributed Chinese isolates from clinical cases. These strains were associated with globally distributed clonal groups linked with confirmed foodborne listeriosis outbreak and sporadic cases. We found that *L. monocytogenes* strains from clonal complex (CC) CC8, CC7, CC9, and CC415 carried most of the adherence and invasive genes. Conversely, CC1, CC2, CC4, and CC6 have the least number of adherence and invasive genes. Additionally, *Listeria* pathogenicity island-1 (LIPI-1), LIPI-2, intracellular survival, surface anchoring, and bile salt resistance genes were detected in all isolates. Importantly, LIPI-3 genes were harbored in CC3, CC224, and ST619 of the Chinese isolates and in CC1, CC4, and CC6 of other worldwide isolates. Notably, Chinese isolates belonging to CC14 carried antibiotic resistance genes (ARGs) against β-lactams (*bla*_TEM-101_ *, bla*_TEM-105_) and macrolide (*ermC*-15), whereas CC7 and CC8 isolates harbored ARGs against aminoglycoside (*aadA10_2, aadA6_1*), which may pose a threat to therapeutic efficacy. Phylogenomic analysis showed that CC8, CC7, and CC5 of Chinese isolates, CC8 (Swiss and Italian isolates), and CC5 and CC7 (Canadian isolates) are closely clustered together and belonged to the same CC. Additionally, CC381 and CC29 of Chinese isolates shared the same genomic pattern as CC26 of Swiss isolate and CC37 of Canadian isolate, respectively, indicating strong phylogenomic relation between these isolates. Collectively, this study highlights considerable clonal diversity with well-recognized virulence and antimicrobial-resistant determinants among Chinese and worldwide isolates that stress to design improved strategies for clinical therapies.

## Introduction

*Listeria monocytogenes* is the genetically heterogeneous species (Kathariou, 2002) implicated in numerous outbreaks of invasive listeriosis reported globally, characterized by septicemia, meningoencephalitis, and maternal–fetal infection leading to abortion (Shoai-Tehrani et al., 2019; Pilmis et al., 2020). The acquisition of this disease is mainly due to the consumption of contaminated food (Lomonaco et al., 2015). Clinically, listeriosis is among the obligatory notifiable diseases in several countries. Although listeriosis is associated with a low incidence rate in humans (Lomonaco et al., 2015), this disease is of great concern due to recurrent outbreaks linked with high mortality and morbidity rates. In China, the incidence of invasive listeriosis is associated with a high case fatality rate (Fan et al., 2019) and high mortality worldwide (EFSA, 2012; Self et al., 2019). Currently, *L. monocytogenes* is composed of four phylogenetic lineages and 13 classified serotypes. In the clinics, serotypes 1/2a, 1/2b, 1/2c, and 4b cause the majority of human listeriosis, which is associated with lineage I and lineage II (Den Bakker et al., 2013).

The ability of *L. monocytogenes* to invade and proliferate within host cells depends on a collection of virulence factors (VFs) at each step of the invasive process during the host–pathogen interaction (Vasquez-Boland et al., 2001; Bergman et al., 2013). The presence of various adhesive and invasive genes promotes adhesion or binding and invasion during infection (Burkholder and Bhunia, 2010; Ghosh and Higgins, 2018). In listeriosis, *Listeria* pathogenicity island-1 (LIPI-1) genes are essential for intracellular growth, multiplication, and further spread to adjacent cells during the infectious cycle (Kocks et al., 1992; Vasquez-Boland et al., 1992; Gouin et al., 1994). Invasion-associated surface protein internalin facilitates adherence and internalization of a host cell. The internalin family representing LIPI-2 particularly internalin A and internalin B (InlA and InlB) plays essential roles in overcoming host barriers (Dussurget et al., 2004). Additionally, listeriolysin S (LLS toxin) representing LIPI-3, which promotes posttranslational modifications, is crucial for its biological and bactericidal activity and host microbiota-related activity (Cotter et al., 2008).

During the infectious cycle, various stress proteins play a crucial role in the early stages of *L. monocytogenes* intracellular growth (Rouquette et al., 1998) by preventing the accumulation of altered proteins, which might be toxic for bacteria under stress conditions (Gaillot et al., 2000). On the other hand, intracellular modulator proteins modulate the interferon response by chromatin remodeling (Lebreton et al., 2011), autophagy evasion (Dortet et al., 2011), and dampening the normal immune response (Gouin et al., 2010), which is crucial for the bacterium survival within the host.

Intracellular survival proteins are necessary for intracellular proliferation (O’Riordan et al., 2003) and contribute to the integrity of *L. monocytogenes* cell wall, swimming motility, and resistance to osmotic stress (Alonzo and Freitag, 2010). Immune evasion proteins such as peptidoglycan modification are critical for bacterial survival (Rae et al., 2011) and conferred the resistance to lysozyme (Boneca et al., 2007). Surface-anchoring proteins are responsible for the maturation of lipoproteins (Reglier-Poupet et al., 2003), and bile salt promotes resisting the acute toxicity of bile and for intestinal persistence (Dusserget et al., 2002).

Recently, whole-genome sequencing (WGS) has proven to be a promising and predictive approach for potential virulence and functional characterization of VFs of *L. monocytogenes* strain (Fox et al., 2016), which would not only help to understand virulence mechanisms or strategies but also potentially help to monitor the risk of causing listeriosis (Reddy and Lawrence, 2014). In the past, despite the spectacular gains in knowledge, the virulence contents or factors of globally distributed clonal groups of *L. monocytogenes* remain poorly addressed. In listeriosis, a number of key VFs played a crucial role in the pathogenesis and survival of *L. monocytogenes* within the host during the infectious process (Dussurget et al., 2014). These VFs need to be investigated in order to better understand and interpret the clinical syndrome of invasive listeriosis for disease treatment and public health surveillance. To identify the virulence contents, resistance profiles, and pathogenic potential and establish the phylogenomic relationship, we have selected all available Chinese invasive isolates as well as global contextual isolates that have a clear link with foodborne listeriosis outbreaks, associated with mortality, and high case fatality rate. Moreover, the comparative investigations on phylogenetic relationships between Chinese and global isolates in relation to VFs and antimicrobial resistance features provide an improved understanding of the global epidemiology of invasive listeriosis caused by *L. monocytogenes*.

## Materials and Methods

### Collection of *L. monocytogenes* Isolates

From the year 2012 until now, 13 confirmed clinical isolates were recovered in China for this investigation. All the bacteria were confirmed as *L. monocytogenes* by a commercial biochemical test (API Listeria, BioMérieux, France). These bacterial isolates were further subjected to serotyping based on a serum agglutination test (NISSEIKEN Co. Ltd., Japan).

A total of 60 global *L. monocytogenes* clinical isolates were selected and analyzed in this study to represent diverse geographical locations: China (n = 24) including 13 new strains in this study as described previously, Canada (n = 25), Switzerland (n = 5), USA (n = 4), and Italy (n = 2). These strains are from blood (n = 37), cerebrospinal fluid (CSF) (n = 7), abortion (n = 7), and stool samples (n = 9) (**Table 1**). The reason for selecting these isolates is that all of them were largely globally distributed epidemic clones (ECs) with virulence features and have been confirmed with the foodborne outbreaks of listeriosis and sporadic cases with high case fatality rates and mortality rates. The information about these clinical isolates can be found in **Table 1** (Farber et al., 2000; Pagotto et al., 2006; Baldry, 2010; Knabel et al., 2012; Gaulin et al., 2014; Reimer et al., 2019; Ragon et al., 2008; Lomonaco et al., 2011; Tasara et al., 2014; Centorame et al., 2015; Tasara et al., 2015; Thomas et al., 2015; Tasara et al., 2016; Zhang et al., 2016; Orsini et al., 2019).

**Table 1:** A list of 60 *Listeria monocytogenes* clinical isolates examined in this study.

S. No	Strain	Serotype	lineage	ST	CCs	Source	Country	References
1	01-5252	1/2a	ll	8	8	blood	Canada	Reimer et al., 2019
2	01-6771	1/2a	ll	8	8	blood	Canada	Reimer et al., 2019
3	02-5993	1/2a	ll	8	8	blood	Canada	Reimer et al., 2019
4	03-5473	1/2a	ll	8	8	blood	Canada	Reimer et al., 2019
5	04-5457	1/2a	ll	8	8	blood	Canada	Reimer et al., 2019
6	08-6997	1/2a	ll	8	8	blood	Canada	Reimer et al., 2019
7	08-7669	1/2a	ll	120	8	blood	Canada	Thomas et al., 2015
8	10-1046	1/2a	ll	8	8	blood	Canada	Ragon et al., 2008
9	10-1047	1/2a	ll	120	8	CSF	Canada	Reimer et al., 2019
10	10-1321	1/2a	ll	8	8	blood	Canada	Reimer et al., 2019
11	88-0478	1/2a	ll	8	8	blood	Canada	Reimer et al., 2019
12	95-0093	1/2a	ll	8	8	blood	Canada	Reimer et al., 2019
13	98-2035	1/2a	ll	8	8	blood	Canada	Reimer et al., 2019
14	99-6370	1/2a	ll	8	8	blood	Canada	Reimer et al., 2019
15	02-6679	4b	l	388	388	stool	Canada	Reimer et al., 2019
16	01-1468	1/2a	ll	120	8	CSF	Canada	Reimer et al., 2019
17	10-4754	1/2a	ll	37	37	CSF	Canada	Gaulin et al., 2014
18	10-0813	1/2a	ll	7	7	blood	Canada	Reimer et al., 2019
19	81-0592	4b	l	1	1	blood	Canada	Knabel et al., 2012
20	81-0558	4b	l	1	1	CSF	Canada	Knabel et al., 2012
21	02-1103	4b	l	1	1	CSF	Canada	Reimer et al., 2019
22	10-0809	4b	l	1	1	stool	Canada	Knabel et al., 2012
23	02-1289	4b	l	1	1	stool	Canada	Reimer et al., 2019
24	10-0933	1/2a	ll	394	415	blood	Canada	Reimer et al., 2019
25	10-0810	1/2b	l	5	5	stool	Canada	Farber et al., 2000
26	L0387	1/2a	ll	8	8	blood	China	This study
27	SHL004	1/2a	ll	8	8	blood	China	Zhang et al., 2016
28	L0386	1/2a	ll	8	8	abortion	China	This study
29	L0457	1/2a	ll	8	8	abortion	China	This study
30	SHL011	1/2a	ll	29	29	blood	China	This study
31	L0382	1/2b	l	87	87	blood	China	This study
32	SHL007	1/2b	l	87	87	blood	China	Zhang et al., 2016
33	SHL012	1/2b	l	87	87	CSF	China	Zhang et al., 2016
34	SHL010	4b	l	2	2	blood	China	Zhang et al., 2016
35	L0456	1/2b	l	5	5	stool	China	This study
36	SHL002	1/2b	l	3	3	blood	China	Zhang et al., 2016
37	SHL008	1/2b	l	3	3	blood	China	Zhang et al., 2016
38	L0383	1/2a	l	91	14	blood	China	This study
39	SHL009	1/2a	ll	91	14	blood	China	Zhang et al., 2016
40	SHL001	1/2a	ll	381	381	stool	China	Zhang et al., 2016
41	SHL013	1/2a	ll	391	89	blood	China	Zhang et al., 2016
42	L0369	1/2c	ll	9	9	abortion	China	This study
43	L0370	1/2b	ll	59	59	abortion	China	This study
44	L0385	1/2a	ll	121	121	abortion	China	This study
45	L0375	1/2b	l	619	ST619	abortion	China	This study
46	L0384	1/2b	l	224	224	blood	China	This study
47	L0381	1/2a	ll	7	7	blood	China	This study
48	SHL005	1/2a	ll	7	7	blood	China	Zhang et al., 2016
49	L0458	1/2a	ll	7	7	abortion	China	This study
50	Lm_17439	1/2a	ll	8	8	blood	Italy	Orsini et al., 2019
51	Lm_hs 2008	4b	l	6	6	CSF	Italy	Centorame et al., 2015
52	LmN1546	1/2a	ll	8	8	blood	Switzerland	Tasara et al., 2014
53	Lm60	1/2a	ll	51	8	blood	Switzerland	Tasara et al., 2014
54	Lm3163	1/2a	ll	26	26	blood	Switzerland	Tasara et al., 2016
55	N2306	4b	l	4	4	blood	Switzerland	Tasara et al., 2015
56	Lm3136	1/2a	ll	18	18	blood	Switzerland	Tasara et al., 2015
57	J2-031	1/2a	ll	394	415	stool	USA	Lomonaco et al., 2015
58	J1-220	4b	l	2	2	stool	USA	Lomonaco et al., 2011
59	J1776	4b	l	6	6	stool	USA	Lomonaco et al., 2011
60	07PF0776	4b	l	4	4	blood	USA	McMullen et al., 2012

Details of serotypes, lineages, sequence types, clonal complexes, source, and country of Listeria monocytogenes clinical isolates.

### Antibiotic Susceptibility Testing

Antibiotic susceptibility of 13 Chinese isolates was assayed using the broth microdilution minimum inhibitory concentrations (MICs) method according to the Clinical and Laboratory Standards Institute (CLSI) breakpoint guidelines (CLSI, 2015). The multiple classes of antimicrobials along with MIC range (µg/ml) used in the assay are as follows: β-lactams [ampicillin (AMP), 0.06–32]; penems [imipenem (IPM), 0.032–16], trimethoprim-sulfamethoxazole [cotri-moxazole (COT), 0.032–16], aminoglycosides [gentamicin (GEN), 0.032–16], tetracyclines [tetracycline (TET), 0.032–16], quinolones [ciprofloxacin (CIP), 0.032–16], phenicols [chloramphenicol (CHL), 0.125–64], and ammonium compounds [benzalkonium chloride (BB), 0.25–128]. Breakpoints for ampicillin and trimethoprim-sulfamethoxazole are as per the CLSI guidelines M45-A3 (CLSI, 2015). Since there are no relevant criteria for tetracycline, chloramphenicol, ciprofloxacin, gentamycin, and ciprofloxacin, the susceptibility results of these antibiotics were interpreted based on breakpoints of *Staphylococcus* spp (CLSI, 2017) as reported previously (Tahoun et al., 2017). *Staphylococcus aureus* ATCC29213 and *Escherichia coli* ATCC 25922 were used as quality control strains.

### Whole-Genome Sequencing and Bioinformatic Analysis

The bacteria were cultured in Brain Heart Infusion (BHI) broth, and genomic DNA was extracted from the *L. monocytogenes* isolates using a commercial kit (Tiangen Biotech Beijing, Co., Ltd.) according to the manufacturer’s protocol. DNA quality and concentrations were analyzed by fluorometer using Qubit dsDNA HS Assay (Thermo Fisher Scientific, United States). For each isolate, paired-end genomic libraries were prepared using Nextera DNA Flex library preparation kit (Tiangen Biotech Beijing, Co., Ltd.). Sequencing was performed employing MiSeq Reagent Kit v2 (2x150bp) on the MiSeq platform (Illumina, United States), the paired and raw reads were trimmed using Trimmomatic (Galaxy version 0.36.6) (Bolger et al., 2014), and then draft genomes were assembled by SPAdes 3.12.0 genome assembler (Bankevich et al., 2012).

Furthermore, genomic sequencing data were retrieved from Genbank, Sequence Read Archive (SRA), and NCBI database. For the downstream bioinformatic analysis, SPAdes v3.12.0 genome assembler was used for genomic assembly, Package Snippy v4.4.4 was used to obtain single-nucleotide polymorphisms (SNPs) alignment, and a phylogenetic tree was constructed by IQ-TREE v1.6.12 with the TVM+F+R3 model. Assembled genomes were submitted to multi-locus sequencing typing (MLST) tool (version 2.3.2) (Carroll et al., 2017), which performs *in silico* analysis to determine the MLST profile. Furthermore, with the aim of identifying the antibiotic resistance genes (ARGs), plasmids, and VFs, the draft genomes of strains were investigated using the software ABRicate (Galaxy version.8) by applying the different types of databases, such as NCBI AMR finder Plus (Feldgarden et al., 2019), [ARG-ANNOT] (Gupta et al., 2014), CARD (Jia et al., 2017), ResFinder (Elbediwi et al., 2020a; Xu et al., 2020), and Plasmidfinder (Elbediwi et al., 2020b; Elbediwi et al., 2020c).

### Statistical and Data Projection Analysis

Data were interpreted using the GraphPad Prism 7 software (GraphPad Software, Inc., USA) and PHYLOViZ software 2.0 using the goeBURST algorithm (Feil et al., 2004) for providing the scalable data integration and visualization for multiple phylogenetic inference methods.

## Results

### Serotypes, Lineages, Sequence Types, and Clonal Complex Analysis

**Figure 1:**
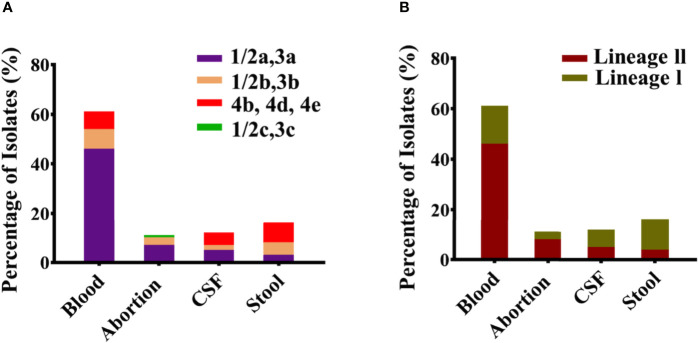
Classification of serotypes and lineages for all clinical *Listeria monocytogenes* isolates. **(A)** Serotypes 1/2a, 1/2b, 1/2c, and 4b representing individually each group/category of blood, abortion, cerebrospinal fluid (CSF), and stool isolates (n = 60). **(B)** Lineage I and Lineage II representing individually each group/category of blood, abortion, CSF, and stool isolates (n = 60).

**Figure 2:**
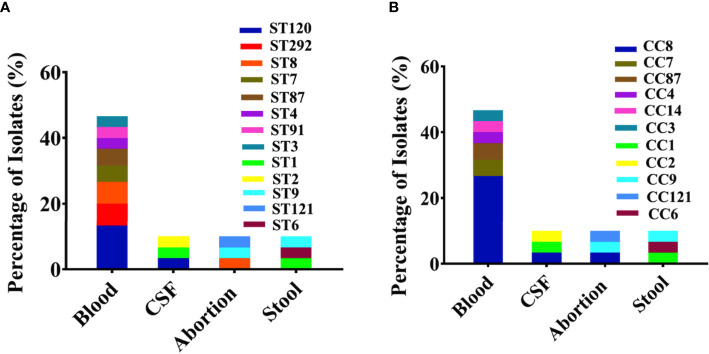
Genetic background with sequence types (STs) and clonal complexes (CCs) for all clinical *Listeria monocytogenes* isolates. **(A)** Major sequence types (STs) representing individually each group/category of blood, abortion, cerebrospinal fluid (CSF), and stool isolates (n = 60) are shown with colors. **(B)** Major CCs representing individually each group/category of blood, abortion, CSF, and stool isolates (n = 60) are shown with colors.

Among the 60 isolates, 61% belonged to blood followed by CSF (12%), abortion (11%), and stool (16%) with predominant serogroup 1/2a (61%) followed by 1/2b (18%), 4b (20%), and 1/2c (1%) that were composed of lineage II (63%) and lineage I (37%) (**Figures 1A, B**). Serum agglutination test showed that among 13 Chinese isolates, seven (54%) belonged to 1/2a, five (39%) belonged to 1/2b, and one belonged to 1/2c. The results of the serum agglutination assay were confirmed by the *in silico* genomic analysis for these 13 Chinese clinical isolates. Overall, these isolates were grouped into 26 different sequence type (STs), 25 were assigned to the clonal complexes (CCs) and one singleton based on querying the MLST database. Among different STs, ST120 (13.33%), ST292 (6.66%), ST8 (6.66%), ST7 (5%), ST87 (5%), ST4 (3.33%), ST91 (3.33%), and ST3 (3.33%) were dominant from blood; followed by ST120 (3.33%), ST1 (3.33%), and ST2 (3.33%) from CSF; and ST8 (3.33%), ST9 (3.33%), ST121 (3.33%), ST1 (3.33%), ST6 (3.33%), and ST9 (3.33%) from stool (**Figure 2A**). Similarly, CC8 (26.65%), CC7 (6.66%), CC87 (5%), CC4 (3.33%), CC3 (3.33%), and CC14 (3.33%) were dominant from blood; followed by CC8 (3.33%), CC1 (3.33%), and CC2 (3.33%) from CSF; and CC8 (3.33%), CC9 (3.33%), CC121 (3.33%), CC1 (3.33%), CC6 (3.33%), and CC9 (3.33%) from stool (**Figure 2B**).

### Genetic Relationship and Phylogenomic Analysis Among Chinese and Worldwide Isolates

**Figure 3:**
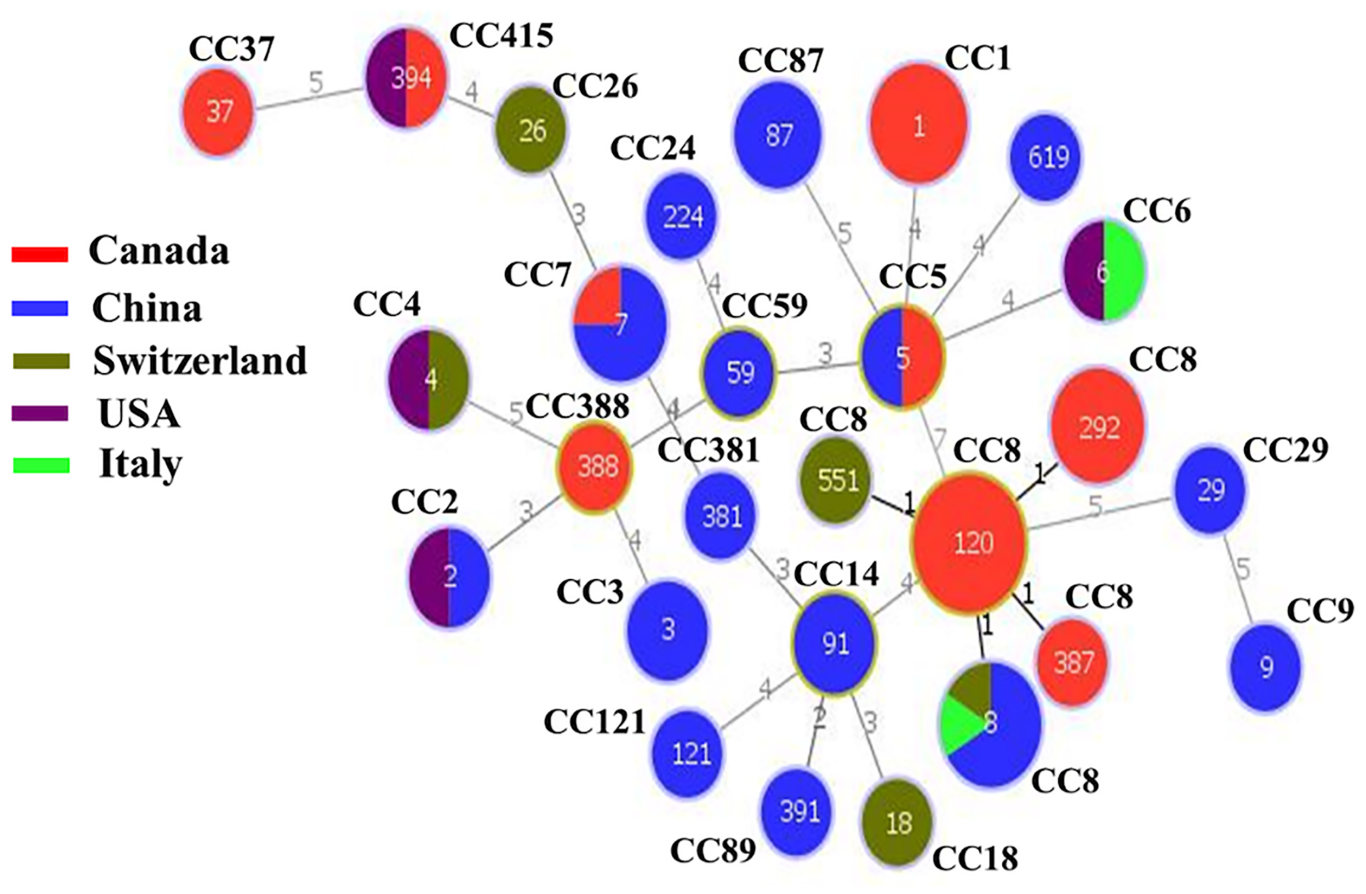
The minimum spanning tree (MST) illustrating the phylogenetic relationship based on sequence types (STs) allelic profiles of clinical strains of worldwide *Listeria monocytogenes* isolates. There are 60 isolates from various countries including China, Canada, Switzerland, USA, and Italy. Each circle represents one ST. The size of the circle is proportional to the number of isolates, and the color within the circle represents country of isolates. Links between the circles are represented according to the number of allelic mismatch between STs.

The minimum spanning tree (MST) showed the relationship among various STs of isolates from different countries. **Figure 3** illustrated that CC8 of Chinese isolates showed the divergence to CC8 of Canadian isolates but closely clustered together with the European CC8 isolates (Italian and Switzerland), while CC5 and CC7 of Chinese and Canadian isolates also showed a close resemblance.

**Figure 4:**
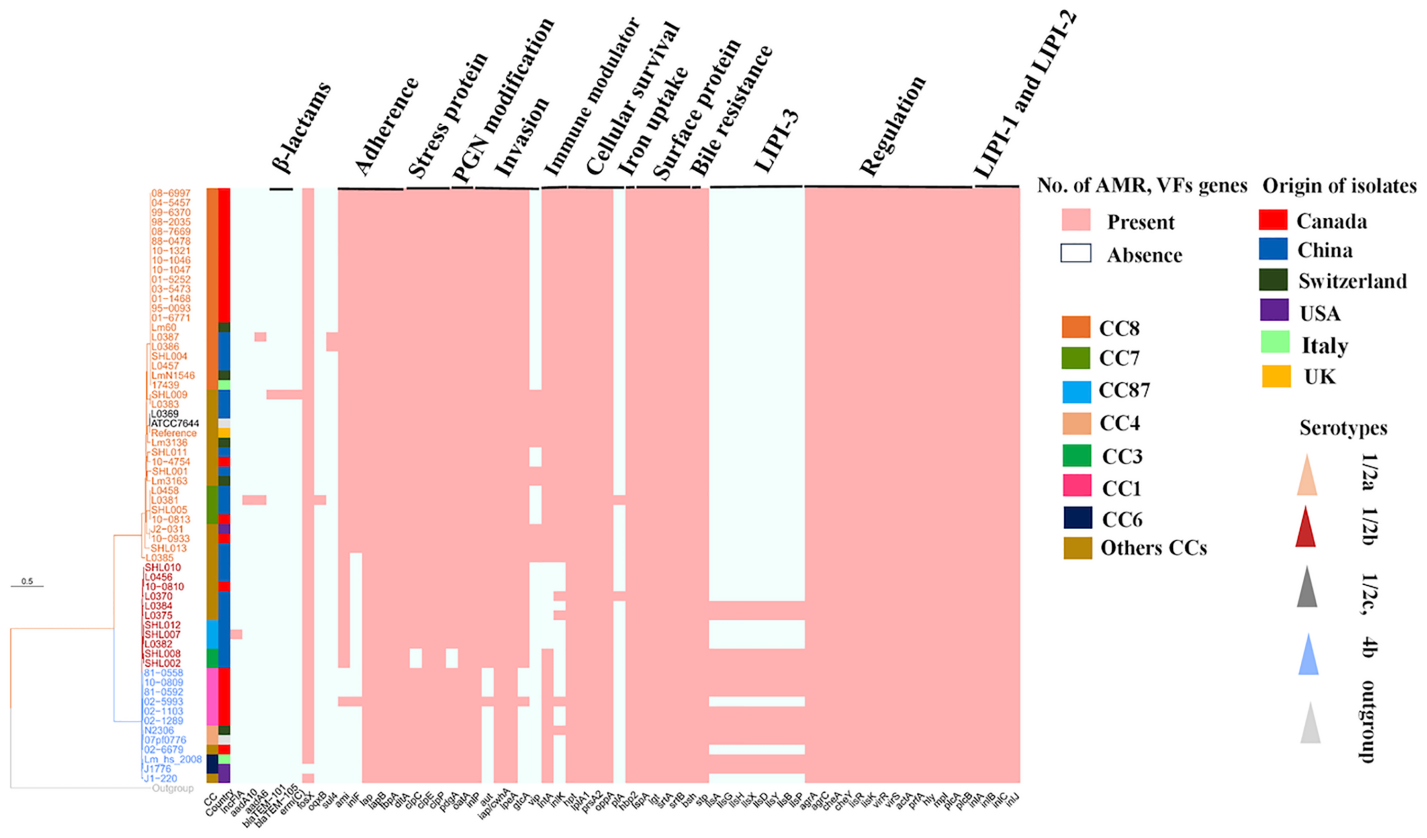
Phylogenomic analysis and heat map composition of antibiotic resistance genes (ARGs) and virulence factors (VFs) of worldwide *Listeria monocytogenes* clinical isolates. There are 60 isolates from different countries including China, Canada, Switzerland, USA, and Italy. Here, 139,505 single-nucleotide polymorphisms (SNPs) were used to construct the maximum likelihood phylogenomic tree for all compared genomes. EGD-e (reference strain), ATCC7644 (control strain), *Listeria inoccua*-CFSAN044836 (out-group strain). Tree scale represents the genetic distance between the isolates used to construct the tree. On the right, there is a heat map about pathogenic composition with clonal complexes (CCs); Plasmid: (*incFIA_1*); ARGs: aminoglycosides: *aadA10_2, aadA6_1*; β-lactams: *bla*_TEM-101_ *, bla*_TEM-105_; macrolide: *ermC-15*; fosfomycin: *fosX_2*; quinolones: *oqx_1*; sulfonamide: *sul4_1*; Adherence genes: *dltA*, *ami*, *inlF, iap, iapB, fbpA*; stress-related genes: *clpC, clpE, clpP*; peptidoglycan modification genes: *pdgA, oatA*; invasion genes: *inlP, aut, iap/cwhA, iapB, ipeA, gtc, vip*; immune modulator genes: *lntA, inlK*; intracellular survival genes: *hpt, ipIA, prsA2, oppA, pIA*; iron uptake genes: *hbp2*; surface protein-anchoring genes: *IspA*, *Igt, srtA, srtB*; bile resistance genes: *bsh*; enzymatic genes: *stp*; listeriolysin S (LLS) toxin (LIPI-3): *llsA, llsG, llsH, llsX, llsD, llsY, llSB, llsP*; regulation genes, *agrA, agrC, cheA, cheY, lisR, lisK, virR, virS*; *Listeria* pathogenicity islands-1 (LIPI-1) genes: *actA, prfA, hly, mpl, plcA, plcB*; *Listeria* pathogenicity islands-2 (LIPI-2) genes: *inlA, inlB, inlC, inlJ*.

A maximum likelihood (ML) phylogenetic tree was constructed with the best model TVM+F+R3 by IQ-tree v1.6.12 using 139,505 SNPs, which were identified from core genome alignments (**Figure 4**) with Snippy v3.1. The phylogenetic tree showed that a high number of isolates with serotype 1/2a (brown color) in the upper clade including L0369 (Chinese isolate belonging to 1/2c, gray color), middle clade (dark brownish red) 1/2b, and lower clade (blue color) 4b (**Figure 4**). Phylogenomic analysis showed that CC8 of Chinese isolates (L0386, L087, SHL004, L0457) has shown a close resemblance to two Swiss isolates (Lm60 and LmN1546) and one Italian isolate 17439 that belonged to CC8. Furthermore, three Chinese isolates (L0458, L0381, and SHL005) from CC7 have shown the same conformity as CC7 of the Canadian isolate (10-0813). Conversely, in one clade, the genome of CC29 of the Chinese isolate showed the same pattern of genes to CC37 of the Canadian isolate (10-4754). Interestingly, these isolates were of serotype 1/2a and composed of lineage II and showed a similar pattern of genomic diversity with the absence of *vip*, *pIA*, and LIPI-3 genes (**Figure 4**).

It has to be mentioned that one Chinese isolate (SHL001) CC381 has shown phylogenetic association with the Swiss isolate (Lm 3163) CC26 that were of serotype 1/2a and lineage II. Both shared similar gene patterns by the absence of *pIA* and LIPI-3 genes. Moreover, CC2 and CC5 of the Chinese isolates (SHL010, L0456) were closely linked to CC5 of the Canadian isolate (10-0810) composed of lineage I with the absence of *vip*, *intA*, *inlK*, *pIA*, and LIPI-3 genes (**Figure 4**).

### Adherence, Invasion Genes, Along With LIPI-1, LIPI-2, And LIPI-3 Genes

The adhesive and invasive genes such as *inlF, ami*, *and aut* were harbored in CC8 (n = 22), CC7 (n = 3), CC415 (n = 2), CC9 (n = 1), and CC14 (n = 2). These strains were serotype 1/2a and comprised lineage II from Canada, USA, and China (**Figure 4**). A similar pattern for the presence of genes was followed by CC26, CC18, CC14, and CC381 from the same serotype 1/2a and lineage ll, while *inlF* and *aut* genes were missing from CC1 Canadian isolates (n = 5) of serotype 4b that was comprised lineage l. Notably, one isolate (02-993) from CC1 showed aberrant features by the presence of these virulence genes (**Figure 4**). In our study, *inlF* was also missing from 33% of the isolates, which belonged to CC2, CC3, CC4, CC5, CC6, CC59, CC87, CC121, CC224, and CC388 that comprised lineage I and lineage II from China, Canada, USA, Switzerland, and Italy. On the other hand, *aut* seems to be missing from 12% of isolates belonging to CC2, CC4, CC6, CC121, and CC388, comprising lineage I and II from China, USA, Switzerland, Italy, and Canada. Moreover, *ami* seems to be absent from 17% of isolates including CC1, CC2, CC6, CC4, and CC388 of serotype 4b that comprised lineage I from Canada, China, Italy, USA, and Switzerland.

LIPI-1 (*plcB, mpl, plcA*, *hyl*, *actA*, *prfA*) and LIPI-2 (*inlA, inlB, inlC, inlJ*) genes were detected in all genomes. Chinese isolates, CC3/ST3 (n = 3), CC224/ST224 (n = 1), and ST619 (n = 1) from serotype 1/2b and worldwide isolates including CC4/ST4 (n = 2), CC6/ST6 (n = 2), and CC1/ST1 (n = 5) of serotype 4b harbored LIPI-3 (*llsA*, *llsG*, *llsH*, *llsX*, *llsD*, *llsY*, *llsB*, *llsP*) genes (**Figure 4**).

### Stress, Immune Modulator, Peptidoglycan Modification, Intracellular Survival, Hexose Phosphate Transporter, Surface Anchoring, and Bile Salt Resistance Genes

Only two Chinese isolates lacked *clpC* gene, while all other isolates harbored the stress-related genes *clpC*, *clpE*, and *clpP*. The presence of immune modulator genes such as *lntA* and *inlK* were harbored in CC8, CC7, CC415, CC9, and CC14 that comprised lineage II. A similar pattern for the presence of genes was followed by CC26, CC18, CC14, CC89, CC415, CC121, and CC381 that comprised lineage II from Switzerland, China, Canada, and USA, whereas these genes were missing from CC2 and CC87 isolates. Additionally, *lntA* gene was missing from 8% of isolates including CC5, CC59, CC224, and CC388 that comprised lineage I from China and Canada, while the *inlK* gene was missing from 16% of isolates including CC6, CC4, CC5, and CC3 that comprised lineage I from the USA, Italy, Switzerland, China, and Canada (**Figure 4**). All the isolates harbored intracellular survival genes *lpIA1*, *oppA*, and *prsA2*, hexose phosphate transporter *hbpt2* gene, surface-anchoring *lspA*, and bile salt hydrolase *bsh* genes (**Figure 4**).

### Antibiotic Resistance Genes and Plasmid Genes

The antimicrobial susceptibility results showed that all the Chinese isolates were 100% sensitive to eight different antibiotics (shown in *Antibiotic Susceptibility Testing* section). The prediction of ARGs was performed based on in-house galaxy format. We have found the ARGs in three Chinese isolates (**Figure 4**). Specifically, one genome of Chinese isolate (CC14/ST91) harbored ARGs to β-lactams (*bla*_TEM-101_, *bla*_TEM-105_) and macrolide (*ermC_15*). Furthermore, an isolate (ST7/CC7) also harbored ARGs to aminoglycosides (*aadA10_2* and *aadA6_1*) and quinolones (*oqxB_1*), while another isolate CC8/ST8 carried *aadA6_2* and *sul4_1* against aminoglycosides and sulfonamides, respectively. Our results showed that only one Chinese isolate (CC87/ST87) harbored *incFIA_1* plasmid gene (**Figure 4**).

## Discussion

*L. monocytogenes* has the potential to cause invasive listeriosis in humans including septicemia, meningoencephalitis, and maternal–fetal infection leading to abortion. The clinical strains analyzed in this study were recovered from systemic investigation and distributed among different regions and associated with clinical outbreaks; therefore, investigating the VFs provided a unique opportunity for understanding the complete genetic makeup of a particular strain.

Overall, these clinical isolates were grouped into 1/2a, 1/2b, 4b, and 1/2c serotypes (**Figure 1A**). Serotype 1/2a was prevalent in the Chinese, Canadian, and Swiss isolates, while 1/2c (L0369 isolate) was identified only in the Chinese isolates, and 4b were mostly associated with the Canadian, USA, and Italian isolates. Serotypes 1/2a and 1/2b were likely to possess greater invasion ability than 1/2c (rarely involved) in listeriosis and may cross the placental barrier (Holch et al., 2013). A study reported that 95% of isolates in listeriosis were of serotypes 1/2a, 1/2b, and 4b (Althaus et al., 2014). Most of the isolates were composed of lineage II, followed by lineage I (**Figure 1B**), which is in accordance with other studies (Chen et al., 2009; Meloni et al., 2009). In this study, most of the clinical isolates belonged to serotype 1/2a that was associated with lineage ll. Additionally, previous studies also witnessed the overrepresentation of lineage ll among clinical isolates. For example, 504 clinical isolates of *L. monocytogenes* recovered for genomic study during 1958–2010 from Sweden belong to lineage ll (Lopez-Valladares et al., 2017), while 52 epidemiological *L. monocytogenes* isolates collected between 1981 and 2011 and 41 isolates from lineage ll were reported as the causative agents for the invasive listeriosis outbreak in Canada (Knabel et al., 2012). Furthermore, a study aiming at the evolutionary relationship of invasive listeriosis of 20 outbreak-associated isolates from the USA showed that the majority of *L. monocytogenes* isolates were from lineage ll (Bergholz et al., 2016). While another investigation showed that 46 invasive listeriosis case outbreaks belong to lineage ll in Austria (Fretz et al., 2010). In China, among 46 clinical strains, 24 strains were found from lineage ll from invasive listeriosis during the study period of 2014–2016 (Zhang et al., 2019).

We have found considerable diversity in STs from clinical sources, which were linked with clinical outbreaks, high case fatality rates, and deaths. For example, the major STs including ST120 (13.33%), ST8 (6.66%), and ST1 (3.33%) (**Figure 2A**) had been associated mainly with Canadian isolates, while ST87 (5%), ST7 (5%), and ST9 (3.33%) were mainly from China; higher prevalence of these STs was also reported by other studies (Maury et al., 2016; Wang et al., 2018; Zhang et al., 2019). Nevertheless, the application of MLST to subtype *L. monocytogenes* has provided updated information regarding the population structure within world regions for clinical isolates (Ragon et al., 2008; Chenal-Francisque et al., 2011; Wang et al., 2012).

The MST of STs of different isolates (**Figure 3**) illustrated that CC8 of Chinese isolates exhibited divergence from Canadian isolates but showed close proximity to European CC8 isolates (Italian and Switzerland). Similarly, CC5 and CC7 of Chinese and Canadian isolates showed a close resemblance. This shows that different strains from a variety of countries might show a similar pattern of genes. The close proximity pattern in relation to distributed genes from different regions also suggests that bacteria may frequently be transported between places of food production, possibly alongside imported raw materials. On the contrary, it is particularly interesting that other genes were conserved among specific CCs, serotypes, and countries.

The presence of adhesive and invasive genes such as *inlF, ami*, and *aut* harbored in CC8, CC7, CC415, CC9, and CC14 composed of lineage II (**Figure 4**). A similar pattern for the presence of genes was followed by CC26, CC18, CC14, CC415, and CC381, which might indicate that these strains have the capacity to cross the blood–brain barrier and fetoplacental barrier. Importantly, the *inlF* gene encodes for a protein that mediates invasion of the brain of the host by binding with vimentin (Ghosh and Higgins, 2018). Additionally, the virulence gene *aut*, which encodes for the *aut* protein, is crucial for the entry of *L. monocytogenes* to the host cell, unaffected by the regulation of *prfA* gene (Cabanes et al., 2004). Similarly, *ami* gene encodes an autolytic amidase with an N-terminal catalytic domain and a C-terminal cell wall-anchoring domain, which has been reported to be involved in the adhesion to eukaryotic cells *via* its cell wall-binding domain (Milohanic et al., 2001).

Conversely, *inlF* and *aut* genes were missing from all Canadian CC1 (n = 5) isolates. Notably, one isolate (02-993) from CC1 showed aberrant features by the presence of these virulence genes. This difference might be due to variation in other genes, as ST relations were based on only seven genes. In our study, *inlF* was also missing from 33% of isolates belonging to CC2, CC3, CC4, CC5, CC6, CC59, CC87, CC121, CC224, and CC388 composed of lineage I and lineage II (**Figure 4**). On the other hand, *aut* seems to be missing from 12% of isolates that belonged to CC2, CC4, CC6, CC121, and CC388 comprising lineage I and II. Moreover, *ami* seems to be absent from 17% of isolates including CC1, CC2, CC6, CC4, and CC388 that comprised lineage I (**Figure 4**). Collectively, these findings may suggest that ST1/CC1, ST2/CC2, ST4/CC4, and ST6/CC6 isolates have lost the crucial genes relating to adherence in the host based on the lack of *inlF* and *aut* genes. The previous investigation reported that CC1 and CC2 have been verified to be strongly associated with clinical origin particularly human central nervous system (CNS) and maternal–neonatal (MN) listeriosis (Maury et al., 2016).

All the isolates harbored adherence and invasive genes of LIPI-1 (*actA*, *prfA*, *hlyA*, *mpl*, *plcA*, *plcB*) and LIPI-2 (*inlA*, *inlB*, *inlC*, *inlJ*) (**Figure 4**). A previous study reported that InlA&InlB helps in binding and invasion (Dramsi et al., 1995) and promotes the entry into the host cell by the process of phagocytosis (Cossart and Toledo‐Arana, 2008). Laterally, the bacteria may further cross the blood–brain barrier and the placental barrier through the hematogenous route. One study (Gouin et al., 2010) reported that InlC dampens the host innate response induced by a pathogen. Furthermore, LLO, a pore-forming toxin that is encoded by HlyA, acts with exo- enzymes (PlcA, PlcB) to facilitate phagosome escape and cell-to-cell spread within the host (Marquis et al., 1995). The master virulence regulator PrfA controls the expression of *hlyA* and *actA* genes (Travier et al., 2013). Intracellular motility is dependent upon the ActA protein, which is essential for the polymerization of host F-actin (Kocks et al., 1992).

In the present study, Chinese isolates of CC224, CC3, and ST619 of serotype 1/2b and worldwide isolates belonged to CC1, CC4, and CC6 of serotype 4b that harbored the LIPI-3 genes (**Figure 4**). These LIPI-3 genes are strongly associated with serotype 4b because of their higher virulence potential by surviving in polymorphonuclear neutrophils (PMNs) (Cotter et al., 2008). These findings further showed the presence of LIPI-3 genes in different CCs belonging to Chinese and worldwide isolates.

Notably, all the isolates harbored stress-related genes, such as *clpP*, *clpE*, and *clpC*. Chinese CC3 isolates (blood, n = 2) lacked crucial virulence gene *clpC* (**Figure 4**). It has to be mentioned here that *clpC* acts synergistically with *clpE* (another stress gene) that is involved in the expression of virulence (Nair et al., 1999), while Gaillot et al. (2001) witnessed that the *clpP* gene is essential for intracellular survival in macrophages and modulates LLO-dependent anti-*Listeria* protection. This showed that CC3 of the Chinese isolates may have lost the crucial genes related to stress.

The presence of immune modulator genes, such as *lntA* and *inlK*, were detected in CC8, CC7, CC415, CC9, and CC14, which were composed of lineage II. The *lntA* gene modulates the interferon responses by playing with chromatin-related mechanism (Lebreton et al., 2011), while the *inlK* gene (Dortet et al., 2011) plays an important role in the escape of autophagy recognition. A similar pattern for the presence of genes was followed by CC26, CC18, CC14, CC89, CC415, CC121, and CC381, while these genes were missing from CC2 and CC87 isolates (**Figure 4**). Additionally, *lntA* genes were missing from 8% of isolates including CC5, CC59, CC224, and CC388 that comprised lineage I, while the *inlK* gene was missing from 16% isolates including CC6, CC4, CC5, and CC3 that comprised lineage I (**Figure 4**).

It is worthy to be mentioned that the peptidoglycan modification genes *pgdA* and *oatA* were harbored in all isolates except CC3 isolates, where the *pgdA* gene was absent; this gene protects *L. monocytogenes* from killing by phagocytes possibly by acetylating the muramic acid residues of peptidoglycan and deacetylates N-acetyl-glucosamine residues (Boneca et al., 2007; Rae et al., 2011), conferring different levels of resistance to antimicrobial peptides and lysozymes. This shows that CC3 Chinese isolates may have lost the crucial genes related to lysozyme, and antimicrobials, which might be trapped by the host defense mechanism during the infection process.

Notably, all the isolates harbored the intracellular survival genes *hpt*, *lpIA1*, and *prsA2* (**Figure 4**). Previous findings reported that the *lpIA1* gene is necessary for intracellular replication, and LpIA1-dependent utilization of host lipoyl peptides enables *L. monocytogenes* cytosolic growth and virulence (Keeney et al., 2007). Additionally, PrsA2-like chaperones were reported to assist in the folding of proteins translocated across the bacterial membrane, whereas these proteins were found to be essential for bacterial viability and relevant to host infection (Alonzo and Freitag, 2010). *L. monocytogenes* possesses hexose phosphate (HP) transporter as a source of carbon and energy that mediates rapid intracellular replication (Chico-Calero et al., 2002).

Moreover, all the isolates carried genes for surface-anchoring *lspA* and bile salt hydrolase *bsh* (**Figure 4**), which help the bacteria to survive under stress encountered within the gastrointestinal tract, including bile. Previous studies reported that LspA is responsible for the maturation of lipoproteins in pathogenesis (Reglier-Poupet et al., 2003), and BSH activity increases under anaerobic conditions, suggesting that anaerobic conditions influence stress responses (Dussurget et al., 2002).

The antimicrobial susceptibility results demonstrated that all the tested isolates (100%) were sensitive to various antibiotics, while the prediction of ARGs showed that three Chinese isolates were resistant (**Figure 4**). Among the Chinese isolates, those of CC14 harbored the resistance gene (*bla*_TEM-101_, *bla*_TEM-105_) to β-lactams and (*ermC*-15) erythromycin. The most common and important mechanism through which bacteria can become resistant against β-lactams is by expressing β-lactamases, for example, extended-spectrum β-lactamases (ESBLs) (Poirel et al., 2007; Queenan and Bush, 2007; Jacoby, 2009). On the other hand, erythromycin resistance is due to the presence of ribosomal RNA (rRNA) methylases, encoded by the *erm* genes (Roberts et al., 1999; Roberts, 2008). CC7 isolate harbored resistance gene against aminoglycoside (*aadA10_2, aadA6_1*) and quinolone (*oqxB_1*). The major encountered aminoglycoside resistance mechanism is due to the modification of enzymes, such as acetyltransferases, acetyltransferases, and phosphotransferases (Wright and Thompson, 1999; Ramirez and Tolmansky, 2010). It has to be mentioned that quinolone resistance may result in either a decreased outer-membrane permeability or mutations of the molecular targets of topoisomerase IV or DNA gyrase enzyme (Hooper, 2000; Ruiz, 2003; Jacoby, 2005; Jiang et al., 2021). The CC8 isolate harbored ARGs against aminoglycoside (*aadA6_1*) and sulfonamide (*sul4_1*). Sulfonamide resistance is raised by mutations in the *folP* gene encoding dihydropteroate synthase (DHPS) enzyme (Skold, 2001; Grape, 2006). These collective findings indicated that Chinese isolates conferred the resistance to important clinical drugs such as β-lactams, aminoglycosides, and erythromycin class; β-lactam class is the primary therapeutic option for human listeriosis together with aminoglycosides (Leong et al., 2016).

Parallelly, *L. monocytogenes* is known to carry several plasmids that often confer resistance to antimicrobial agents or even increased stress resistance (Kuenne et al., 2013). The analysis of the plasmids identified that none of the isolates in this study carried a plasmid except only CC87 Chinese isolate harbored plasmid *incFI_A* gene (**Figure 4**). Taken together, these findings suggest that Chinese clones are somewhat more resistant due to the presence of resistance genes as described previously (Elbediwi et al., 2019; Wang et al., 2019; Elbediwi et al., 2020c; Paudyal et al., 2020) as compared to other worldwide isolates that may pose a significant threat to the public. Therefore, these findings call for prompt action and stress for adapting a one-health approach (Zhou et al., 2020) for formulating the policies on microbiological safety (Paudyal et al., 2018).

We have found through phylogenetic analysis that Chinese and Swiss isolates are closely related and clustered together in the same clade and belonged to the same CC (**Figure 4**). For example, three CC8 Chinese isolates showed a close resemblance to two Swiss isolates and one Italian isolate (CC8), CC7 of three Chinese isolates phylogenetically related to CC7 of one Canadian isolate, and one CC5 showed a similar pattern of genes as CC5 of the Canadian isolate. On the other hand, CC381 of the Chinese isolates shared a similar pattern with CC26 (Swiss isolate), and CC29 was closely linked with CC37 of the Canadian isolate. This might suggest the possible”antigen switching” from one region to another with the opportunity to adapt and acquire resistance, persistence, and virulence under diverse geographical locations.

## Conclusion

These findings demonstrated that CC8, CC7, CC9, and CC415 contained virulence and invasive genes *inlF*, *ami*, and *aut*, while CC1, CC2, CC4, and CC6 lacked these genes. Chinese isolates that belonged to CC8, CC7, CC5, CC81, and CC29 shared similar genomic patterns with other worldwide clones. Furthermore, LIPI-3 genes were harbored in CC3, CC224, and ST619 genomes of the Chinese isolates and CC1, CC4, and CC6 of other worldwide isolates. LIPI-1 and LIPI-2 as well as genes involved in intracellular survival, surface anchoring, and bile salt hydrolase were harbored in all examined isolates. Additionally, the presence of various ARGs and plasmid genes in Chinese clones may pose a serious risk to public health. In a nutshell, uncovering the diversity of VFs and features of antimicrobial resistance of *L. monocytogenes* for better clinical therapies for invasive listeriosis could improve the patient outcome. The considerable clonal and genomic diversity along with well-recognized virulence-associated genotypes and antimicrobial-resistant determinants was shared among Chinese and worldwide isolates that stress to design and improve the strategies of clinical therapies. Apart from these, factors other than enhanced virulence such as survival, growth characteristics in food-associated environment, and host immune status should also be considered in further investigation.

## Data Availability Statement

The datasets generated for this study can be found in NCBI Bioproject number PRJNA688596.

## Ethics Statement

Written informed consent was obtained from the individual(s) for the publication of any potentially identifiable images or data included in this article.

## Author Contributions

Conceptualization: MY and SX. Investigation: DS, TA, and WC. Validation: DS and WC. Data analysis: TA and HP. Writing-original draft preparation: TA. writing-review and editing: MY and TA. Project administration and funding acquisition: MY and SX. All authors contributed to the article and approved the submitted version.

## Funding

This work was supported by the National Program on Key Research Project of China (2019YFE0103900) as well as the European Union’s Horizon 2020 Research and Innovation Programme under Grant Agreement No. 861917–SAFFI, Zhejiang Provincial Natural Science Foundation of China (LR19C180001), Zhejiang Provincial Key R&D Program of China (2021C02008 and 2020C02032), and Opening Fund of Key Laboratory of Microorganism Technology and Bioinformatics Research of Zhejiang Province (2017E10010).

## Conflict of Interest

The authors declare that the research was conducted in the absence of any commercial or financial relationships that could be construed as a potential conflict of interest.

## Publisher’s Note

All claims expressed in this article are solely those of the authors and do not necessarily represent those of their affiliated organizations, or those of the publisher, the editors and the reviewers. Any product that may be evaluated in this article, or claim that may be made by its manufacturer, is not guaranteed or endorsed by the publisher.

## Supplementary Material

Supplementary Table 1 The name of repositories along with accession number of clinical isolates.
