# Supplementary material for: Genomic Determinants of Pathogenicity and Antimicrobial Resistance for 60 Global Listeria monocytogenes Isolates Responsible for Invasive Infections
Source: Front Cell Infect Microbiol. 2021 Oct 27;11:718840. doi: 10.3389/fcimb.2021.718840 (PMC8579135; doi:10.3389/fcimb.2021.718840)
Supplement: Supplementary Table 1 — The name of repositories along with accession number of clinical isolates. [file DataSheet_1.docx]

Supplementary material

Table1.The name of repositories and accession number of clinical isolates

**Table 1**

| **S. No** | **Strain ID** | **Accession number** | **Bio project number** |
| --- | --- | --- | --- |
| 1 | L0369 | SAMN17182357 | PRJNA688596 |
| 2 | L0370 | SAMN17182358 | PRJNA688596 |
| 3 | L0375 | SAMN17182359 | PRJNA688596 |
| 4 | L0381 | SAMN17182360 | PRJNA688596 |
| 5 | L0382 | SAMN17182361 | PRJNA688596 |
| 6 | L0383 | SAMN17182362 | PRJNA688596 |
| 7 | L0384 | SAMN17182363 | PRJNA688596 |
| 8 | L0385 | SAMN17182364 | PRJNA688596 |
| 9 | L0386 | SAMN17182365 | PRJNA688596 |
| 10 | L0387 | SAMN17182366 | PRJNA688596 |
| 11 | L0456 | SAMN17182367 | PRJNA688596 |
| 12 | L0457 | SAMN17182368 | PRJNA688596 |
| 13 | L0458 | SAMN17182369 | PRJNA688596 |
